# Outcomes of organ‐sparing surgery for adult testicular tumors: A systematic review of the literature

**DOI:** 10.1002/bco2.77

**Published:** 2021-02-23

**Authors:** Jesse Ory, Udi Blankstein, Daniel C. Gonzalez, Aditya A. Sathe, Joshua T. White, Carlos Delgado, John Reynolds, Keith Jarvi, Ranjith Ramasamy

**Affiliations:** ^1^ Department of Urology Miller School of Medicine University of Miami Miami FL USA; ^2^ Department of Surgery Division of Urology University of Toronto Toronto ON Canada; ^3^ College of Medicine University of Tennessee Health Science Center Memphis TN USA; ^4^ Department of Urology Dalhousie University Halifax NS Canada; ^5^ School of Medicine and Health Science Tecnologico de Monterrey Monterrey Mexico; ^6^ Department of Health Informatics Miller School of Medicine University of Miami Calder Memorial Library Miami FL USA

**Keywords:** germ cell, Leydig cell tumor, macroscopic, microscopic, organ‐sparing, testis, testicular neoplasms, testis‐sparing surgery

## Abstract

**Objective:**

To perform a systematic review on the effects of testicular sparing surgery (TSS) on the oncological, functional, and hormonal outcomes of adults with testicular tumors.

**Methods:**

A literature search was performed after PROSPERO registration (CRD42020200842) and reported in compliance with Preferred Reporting Items for Systematic Reviews and Meta‐Analyses (PRISMA) methods. We conducted a systematic search of Medline (Ovid), Embase, Cochrane CENTRAL, CINAHL, Scopus, Web of Science, ClinicalTrials.gov, and the WHO/ICTRP from inception to November 20, 2020. Manuscripts and published abstracts were included if they involved testis‐sparing surgery (TSS) and contained data on any outcomes related to fertility, hormonal parameters, or oncological control, or if they evaluated surgical technique.

**Results:**

Our initial search yielded 3,370 manuscripts, with 269 of these screened for full‐text eligibility. After our exclusion criteria were applied, 32 studies were included in the final analysis. Oncological outcomes were obtained from 12 studies (average follow‐up 57.8 months), functional data from 26 studies (average follow‐up 49.6 months), fertility information from 10 studies (average follow‐up 55.8 months), and data on nonpalpable tumors from 11 studies (average follow‐up 32.1 months). Oncological control appears to be excellent in studies that reported these outcomes. Presence of germ cell neoplasia in situ was controlled with adjuvant radiation in nearly all cases. Functional outcomes are also promising, as development of primary and compensated hypogonadism was rare. Semen parameters are poor preoperatively among men with benign and malignant testis tumors, with occasional decline after TSS. Frozen section analysis at the time of surgery appears to be very reliable, and the majority of nonpalpable tumors appear to be benign.

**Conclusions:**

TSS is a safe and efficacious technique with regards to oncological control and postoperative hormonal function based on retrospective, noncontrolled studies. TSS avoids unnecessary removal of benign testicular tissue, and should be given serious consideration in cases of nonpalpable, small tumors under 2 cm. In cases of malignancy, TSS can safely avoid anorchia in men with bilateral tumors and in men with solitary testicles. The use of the operating microscope, while theoretically promising, does not necessarily lead to better outcomes, however data are limited.

## 1 INTRODUCTION

Germ cell and sex cord testicular tumors (Leydig/Sertoli/Granulosa cell) continue to be a relatively common GU malignancy in young men. Due to an exceptionally high cure rate for testis malignancy, we need to consider issues with survivorship such as fertility, testosterone production, and quality of life. This is especially pertinent given the young age at which most men are diagnosed and the long‐term surveillance protocols are employed. Early perspectives and oncological dogma necessitate the use of radical inguinal orchiectomy as the gold standard treatment for men with testicular masses. There is evidence to suggest that even only undergoing a radical orchiectomy, without subsequent chemotherapeutics or radiotherapy, can cause hormonal and fertility dysfunction such as compensatory Luteinizing Hormone (LH) production and deterioration of semen analysis parameters.^1^, ^2^

The availability and technical improvements of imaging modalities have increased the identification of small, often histologically benign and slow growing testis tumors.^3^, ^4^ The incidence of these nonpalpable tumors can be as high as 7.4%, with a range between 10 and 15 mm.^5^ The management of these lesions is evolving, with some centers offering active surveillance with serial ultrasound examinations as an initial approach.^6^ Moreover, recent studies are beginning to explore the use of ultra‐sensitive mRNA biomarkers to guide more accurate diagnosis, further questioning our current management pathways.^7^

Testis‐sparing surgery (TSS) can be a viable option for men with nonpalpable, sub centimeter, and/or bilateral testicular tumors. Additionally, men with functional or anatomic solitary testicles have also been excellent candidates for TSS. Indeed, most urological societies have embraced this method of organ‐sparing as part of their clinical guidelines.^8^, ^9^, ^10^ Intraoperative ultrasound probes and the use of the operative microscope have also been introduced as additional technical tools to improve the outcomes and decrease the total amount of testicular tissue excised.^11^, ^12^

While it is clear that TSS can prevent overtreatment of benign tumors, ample evidence suggests that TSS is important for maintaining sperm and testosterone production after surgery as these parameters can be abnormal prior to surgery. In the past decade, there has been significant accrual of evidence examining this topic. The aim of this paper is to systematically review the most up‐to‐date evidence with regards to the use of TSS in select scenarios. We hope these data can assist clinicians and surgeons so that they can have informed discussions with their patients with regards to optimal management.

## 2 METHODS

The search strategy was developed by two investigators (J.O. and J.R), and was reviewed using the Peer Review for Electronic Search Strategies (PRESS) tool.^13^ The search strategy was written for Ovid Medline and translated using each database's syntax, controlled vocabulary, and search fields. MeSH terms, EMTREE terms, and text words were used for the concepts of testicular tumors, organ‐sparing surgery, and their synonyms. We searched Ovid Medline (Including Epub‐Ahead‐of‐Print, In‐Process & Other Non‐Indexed Citations and Daily, 1946 to November 20, 2020), Embase (Elsevier, Embase.com, 1947‐present), Cochrane CENTRAL (Cochrane Library, Wiley, no date limit), CINAHL Plus (EBSCO, 1937 to present), Scopus (Elsevier, 1788‐present), and the Web of Science platform (Clarivate: Science Citation Index Expanded (SCI‐EXPANDED)––1945‐present; Social Sciences Citation Index (SSCI)––1956‐present; Arts & Humanities Citation Index (A&HCI)––1975‐present; Conference Proceedings Citation Index‐Science (CPCI‐S)––1990‐present; Conference Proceedings Citation Index‐Social Science & Humanities (CPCI‐SSH)––1990‐present; Emerging Sources Citation Index (ESCI)––2015‐present; KCI‐Korean Journal Database 1980‐present; Russian Science Citation Index 2005‐present; SciELO Citation Index 2002‐present). The SR‐Accelerator Polyglot Search Translator tool was used in part to aide in converting the original search to run in other databases.^14^ An initial, simpler search was also run in Scopus and PubMed prior to the development of the final search strategies and results from these studies were also screened. We searched trials registry Clinicaltrials.gov and the World Health Organization International Clinical Trials Registry Platform (ICTRP), for trials with reported results. Conference abstracts were included in Embase, Scopus, and Web of Science searches. We also reviewed the studies included in two systematic reviews on related topics.^15^, ^16^ No language, date, or other limits were applied. We searched all databases on November 20, 2020. For full search strategies, see online Appendix. All database records were downloaded to EndNote X9,^17^ then uploaded to Covidence web‐based software^18^ for deduplication, screening, and full‐text evaluation. We did not contact any study authors, manufacturers, or other experts. We checked the Retraction Watch database for retractions or corrections of studies selected for inclusion. Our inclusion criteria included adult men over the age of 18 who underwent testis‐sparing surgery for a testicular tumor and reported on any oncological or functional outcome. After our search was completed, two authors (D.G. and J.O.) independently performed an initial abstract screen. Following this, two authors (D.G. and A.S) independently performed a full‐text review with any disputes resolved by a third author (J.O) (Figure 2). Sources of funding were investigated in these studies but were not found for any of the included papers.

D.G, J.W, C.D, and A.G extracted data from the included articles. Extracted variables included study characteristics (eg, Author, year of publication, population, adjuvant therapy, tumor type, and tumor size) and outcomes of interest (eg, Testosterone, sperm parameters, other hormonal levels, and recurrence‐free interval). Relevant data were extracted into a Microsoft Excel database. A meta‐analysis of our data was not able to be conducted due to heterogeneity in study designs, outcomes, and populations of our included studies. Because of this, only descriptive analyses were performed.

## 3 RESULTS/DISCUSSION

### 3.1 Study selection

**FIGURE 1:**
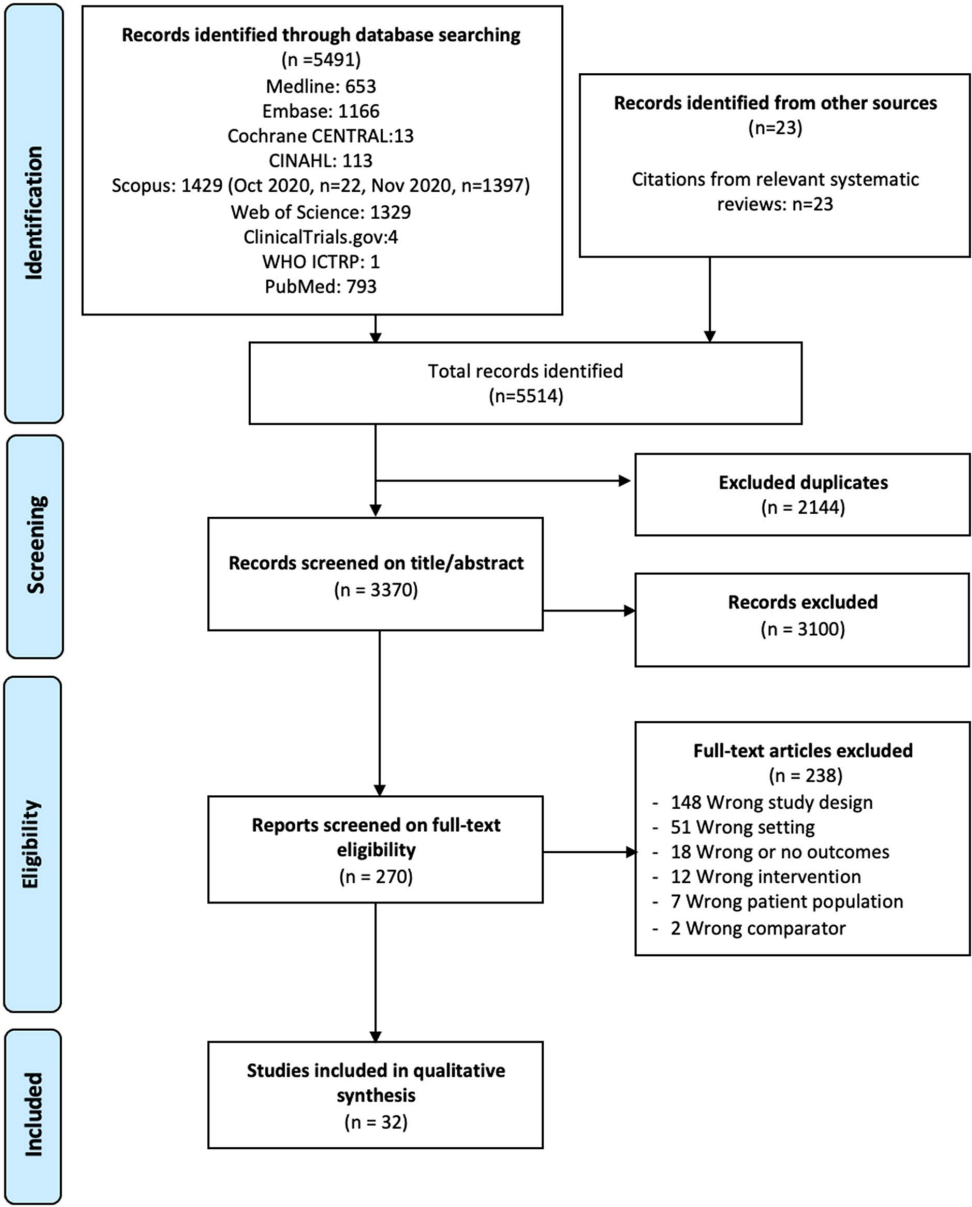
Preferred Reporting Items for Systematic Reviews and Meta‐Analyses (PRISMA) flowchart of screening and selection procedure

Our search identified 5514 records. After duplicated were filtered, we had 3370 studies to screen. After title and abstract screening of these, 270 manuscripts remained for full‐text review. Of these 270 articles, 32 fulfilled the study criteria and were included for further evaluation. The primary cause for exclusion included wrong study design (148), wrong setting (51), wrong/no outcome (18), and wrong intervention (12). Figure 1 describes the process of study inclusion.^19^

### 3.2 Surgical approach

**FIGURE 2:**
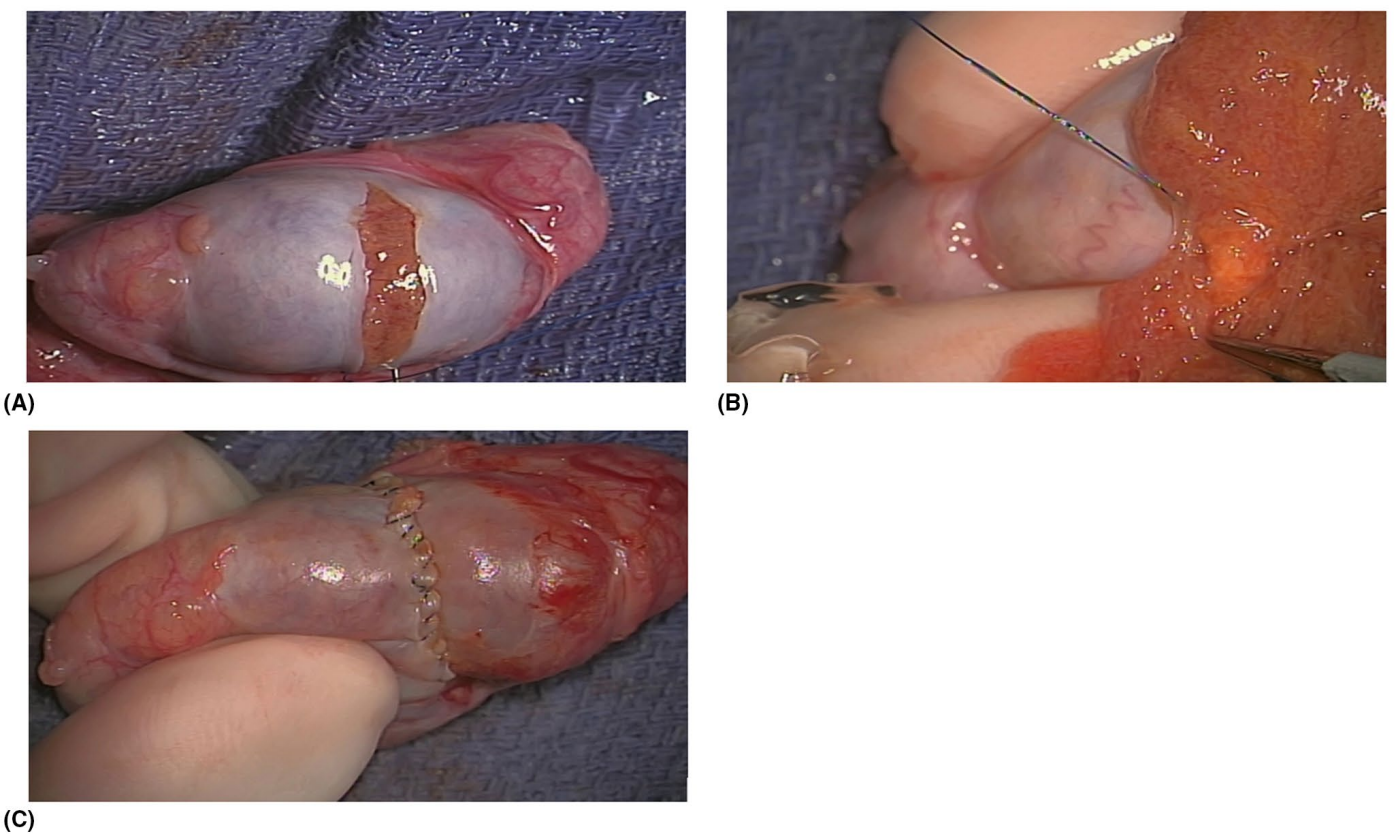
Clockwise from top left: (A) a transverse incision in the tunical albuginea to expose the testicular parenchyma. (B) After ultrasound detection, the yellow Leydig cell tumor is exposed here with a 4k 3D Orbeye Microscope. (C) Closure of the tunica albuginea with 5‐0 prolene running suture

A standard inguinal approach is recommended in the event a radical orchiectomy needs to be performed, thereby avoiding scrotal violation.^20^, ^21^ Early clamping of the cord prior to delivery of the testicle into the operative field has been advocated to decrease the risk of tumor spread from manipulation of the affected testicle, but this long‐held practice holds little basis in literature. In the largest series by Leonhartsberger et al., a non‐clamping approach was utilized in 65 patients for both radical orchiectomy and TSS. They found that all patients were free of disease at a median follow‐up of 52.5 months (range 3‐107 months).^22^ If TSS is being performed, after delivery of the testicle, a transverse incision of the tunica albuginea is recommended in order to identify an avascular plane^23^, ^24^ (Figure 1). If clamping is to be performed, the benefits of cold or warm ischemia are still under debate.^22^ Ultrasound guidance may be beneficial, particularly in cases of nonpalpable lesions.^20^ A microsurgical approach with an operating microscope can be used if equipment is available and surgeon has the skillset (Figure 2). Ultimately, because each individual surgical variation has little to no functional or oncological‐based evidence, we believe that the operative method chosen should be determined by the surgeon's preference.

### 3.3 Microsurgical vs Macrosurgical

The majority of authors described a macrosurgical approach with ultrasound (US) guidance for tumor localization. Surgical loupes were used in two studies.^25^, ^26^ There were four studies which evaluated a microsurgical approach.^23^, ^24^, ^27^, ^28^ There were no studies directly comparing the outcomes of a microsurgical and macrosurgical approach. Magnification varied between 10× and 25× between the microsurgical cases.^23^, ^24^, ^27^, ^28^ The most common indication for a microsurgical approach was a nonpalpable tumor identified on US. The other indication was concurrent testicular sperm extraction (TESE) in an azoospermic man for assisted reproductive techniques that had incidental testicular lesions identified on US. All studies^29^ that we identified for this review included some detail on the surgical approach.

The largest series of microsurgical testis sparing cases by De Stefani et al. included 23 patients.^28^ In this retrospective study, nonpalpable tumors with negative tumor markers were routinely treated with testis‐sparing surgery (TSS) using a microsurgical approach. The mean size of lesion was 16.5 mm ± 8.7 mm. One patient required an additional surgery 5 years from the first, after normal testicular tissue was reported on initial pathology. The repeat surgery revealed seminoma, requiring radical orchiectomy. There were no patients with progression of disease in this group. All patients were disease free and had normal scrotal US findings after a mean follow‐up of 35 ± 25 months. One patient did develop clinical hypogonadism postoperatively though objective hormone levels were not available.

Authors using a microsurgical approach describe potential benefits being increased preservation of normal parenchyma and decreased risk of vascular injury to the tunica albuginea with a theoretical decreased risk of hypogonadism, testicular atrophy, and infertility.^23^, ^28^ We were unable to identify any meaningful differences from a functional or oncological standpoint between patients undergoing microsurgical and macrosurgical techniques due to the small number of reported series. A microsurgical approach does appear to be safe in appropriately selected patients, while the best outcomes for each surgeon will likely be based on their individual experience and preference.

### 3.4 Hormonal changes after testis‐sparing surgery

Changes in reproductive hormone levels have been well documented after radical orchiectomy, with and without adjuvant therapy. Typically, luteinizing hormone (LH) and follicle stimulating hormone (FSH) increase, while most series show either a reduction in testosterone, or at minimum, an increase in rates of compensated hypogonadism (normal testosterone and elevated LH), even in those with orchiectomy alone.^30^, ^31^, ^32^ Rates of compensated hypogonadism (elevated LH with normal T) are much higher in men with solitary testis of any cause,^29^ which by itself carries a risk of significant negative long‐term effects.^33^

Due to these outcomes and associated risks, it is reasonable to attempt to reduce the amount of testicular parenchyma that is removed, thus avoiding subsequent changes in reproductive hormones. We identified 22 studies that reported hormonal outcomes following TSS (Tables 1 and 2). The largest series to date to describe the hormone levels followed 101 men for a median of 80 months after testis‐sparing surgery for a malignant tumor. They reported a 9.7% rate of hypogonadism following TSS, in men with a mean tumor diameter of 15 mm. Most men in this series (79%) underwent adjuvant radiation with 18 Gy.^34^ The largest series reported thus far that describes both preoperative and postoperative testosterone levels found both to be “normal” with few quantitative data in 49 men, and a tumor diameter of less than or equal to 1.5 cm.^12^

**TABLE 1:** Series reporting on functional hormonal changes in benign testicular tumors

First author (Year)	(#) of benign tumors	Mean tumor diameter (mm)	Preoperative analysis			Postoperative analysis		T (ng/dL)	TRT?	Follow‐up (mo)
			LH (mIU/mL)	FSH (mIU/mL)	T (ng/dL)	LH (mIU/mL)	FSH (mIU/mL)			
Keske (2019)^63^	**(10)**	14.6 ± 120.5	NR	NR	453 ± 41	NR	NR	385 ± 43	1	47.2 ± 22.5
	LCT(1)									
	Adenomatoid(2)									
	EC (3)									
	Ischemic infarct (1)									
	Sperm granuloma (1)									
	Tunica albuginea cyst (1)									
	Hyaline changes without tumor (1)									
Pozza (2019)^39^	**(40**) LCT	NR	5.8 (2.7‐7.2)*	4.9 (2.4‐32.1)*	577 (375‐715)*	5.5 (3.6‐6.9)*	6.8 (5.3‐24.22)*	539 (260‐741)*	22	60 (6‐120)
Lagabrielle (2018)^64^	**(24)**	8.5(4.7‐10)*	41.3 (6.6‐11.1)*	22.3 (17.4‐32.8)	453 (332.5‐602.3) *	NR	NR	NR	3	26 (8‐32)
	LCT (23)									
	Scar tissue (1)									
Bozzini (2013)^65^	**(22)** LCT	11.4 (5‐31)	4/22 High	4/22 High	720	NR	NR	530	NR	180 (77‐290)
Steiner (2003)^44^	**(18)**	11.5 (3‐24)*	NR	NR	NR	NR	NR	446 (310‐600)	NR	35.7 (12‐91)
	LCT (10)									
	SCT (2)									
	EC (3)									
	Fibrotic pseudotumor (2)									
	Adenomatoid tumor (1)									
Carmignani (2007)^66^	**(22**) LCT	11.4 (5.0‐31.1)	NR	NR	720	4/22 High	4/22 High	513	NR	47 (1‐230)
Hallak (2009)^24^	**(4**) LCT	6.7 ± 1.8	NR	9.6 ± 5.3	438 ± 146	NR	NR	“Normal” 367‐195	0	NR
Liu (2015)^67^	**(11)**	NR	5.0 ± 2.1	8.5 ± 3.57	401 ± 159	4.7 ± 2.0	8.7 ± 2.9	406 ± 147	0	31.7 ± 15.8
	EC (6)									
	Sertoli cell (3)									
	Mixed sex cord (2)									
Claahsen‐van der Grinten (2007)^68^	6 CAH & TART	NR	8.9 ± 15.0	15.7 ± 20.5	490 (205‐1067)	12.01 ± 14.9	21.3 ± 20.4	366 (37‐ 663)	2	22
Egan (2020)^69^	**12**	10 (6‐14)	NR	NR	384 (246‐503)	NR	NR	348 (312‐709)	0	13.7 (1.8‐25.4)
	LCH (6)									
	EC (1)									
	Calcification (3)									
	SCT (2)									
Gentile (2013)^26^	**13**	10.5 ± 3.1	NR	13/ 13 “Normal”	13/13 Normal	NR	13/13 Normal	13/13 Normal	0	19.2 ± 11.5
Lawrentschuk (2011)^70^	**10**	7.5 (3‐15)	NR	NR	10/10 “Normal”	NR	NR	10/10 “Normal”	1	68.4 (12‐148)
	SCT(2)									
	EC (2)									
	Hematoma (2)									
	Fibrosis(2)									
	Benign mass (1)									
Zu'bi (2019)^71^	**9** LCT	15 ± 10.8	NR	NR	4/6 Elevated	2/4 Undetectable	2/4 Undetectable	1/6 Elevated	NR	31.8 ± 26.3
						2/ 4 Normal	2/4 Normal			
Leonhartsberger (2014)^22^	**37**	8.5 (3‐20)	NR	NR	NR	NR	NR	24/24 “Normal”	0	63 (10‐120)
	LCT (27)									
	EC (5)									
	Fibrous/bone metaplasia (5)									

Note

CAH, congenital adrenal hyperplasia; EC, epidermoid cyst; FSH, follicle stimulating hormone; LCT, Leydig Cell tumor; LCH, Leydig cell hyperplasia; LH, luteinizing hormone; SCT, Sertoli Cell tumor; T, testosterone; TART, testicular adrenal rest tumors; TRT, testosterone replacement therapy.

* Values are expressed as median and interquartile ranges (IQR).

**TABLE 2:** Series reporting on functional hormonal changes for malignant testicular tumors

First author (Year)	No. of malign tumors	Mean tumor diameter (mm)	Adjuvant therapy?	Local recurrence?	Preoperative analysis			Postoperative analysis			Follow‐up (mos)
					LH (mIU/mL)	FSH (mIU/mL)	T (ng/dL)	LH (mIU/mL)	FSH (mIU/mL)	T (ng/dL)	
Keske (2019)^63^	**(3)**	14.6 ± 120.5	12% RT	1/3	NR	NR	247 ± 87	NR	NR	153 ± 37	47.2 ± 22.5
	Seminoma (2)		10% chemo								
	Embryonal carcinoma (1)										
Lagabrielle (2018)^64^	**8**	7.6 (4.4‐9)	2/8 RT	1/8	NR	28 (17.1‐37.8)	580 (540‐620)	NR	NR	NR	26 (8‐32)
	Seminoma (7)										
	1 Teratoma (1)										
Steiner (2003)^44^	**12**	17.1 (6‐30)	2/12	2/12	NR	NR	NR	NR	NR	386 (40‐630)	59.8 (10‐105)
	Seminoma (8)										
	LCT (2)										
	Mixed GCT (2)										
	Fibrotic pseudotumor (1)										
Lawrentschuk (2011)^70^	**17**	14 (3‐27)	1/27	0/27	NR	NR	17/17 “Normal”	NR	NR	17/17 “Normal”	85.2 (12‐169)
	Seminoma (11)										
	Non‐Seminoma (3)										
	Teratoma (2)										
	Mixed GCT (1)										
Weissbach (1995)^25^	**14**	16.0 (6‐30)	14/14 RT	0	NR	NR	380	NR	NR	465	37.9 (20‐108)
	Nonseminoma (8)										
	Seminoma (6)										
Heidenreich (2001)^47^	**52**	15 (3‐50)	46/52 RT	4	NR	NR	420 (390‐440)	NR	NR	310 (290‐340)	91 (3‐191)
	Seminoma (34)										
	Embryonal carcinoma (10)										
	Teratoma (14)										
	Mixed GCT (11)										
Mearini (1996)^72^	**1**	NR	NR	NR	NR	NR	1,090	NR	NR	390	46
	GCT										
Kazem (1999)^73^	Seminoma (2)	17.5 ± 3.5	2/2	0	9.75 ± 8.5	19.3 ± 14.5	397 ± 158	23.7 ± 7.0	57.5 ± 1.6	220 ± 70	43
Wren (2020)^74^	**9**	9 (5‐18)	NR	NR	NR	NR	434 ± 245	NR	NR	267 ± 92	84
	Seminoma (6)										
	Non‐Seminoma (3)										
Ferretti (2014)^75^	**20**	11.6 ± 1.5	7/20	3/20	4/5 “normal”	19.4 ± 19.5	571 ± 45	NR	NR	450 ± 57	42.7
	Seminoma (11)										
	Non seminoma (9)										
Bojanic (2015)^48^	**25**	12.25	0	7/24	NR	25/25 “Normal”	25/25 “Normal”	NR	2 High** (23/25 Normal)	25/25 “Normal”	51 (7‐178)
	Seminoma (16)										
	Non‐Seminoma (9)										
Heidenreich (2006)^34^	**101**	15 (5‐30)	80/101	6/80	NR	NR	6/101 “Low”	NR	NR	84/101 “Normal”	80 (4‐191)
	Seminoma (57)									16/101 “Low”	
	Embryonal carcinoma (20)										
	Teratoma (15)										
	Mixed GCT (9)										

Note

Values are expressed as median and Interquartile range (IQR).

In most series that document hormone levels after TSS for benign lesions, in which adjuvant therapy is unnecessary, subsequent testosterone deficiency is rare (Table 1).

In some series, postoperative hormone levels were not available, yet prescription of testosterone therapy was used as a clinical indicator of hypogonadism. A recent meta‐analysis on TSS revealed 7.1% of patients received testosterone therapy following treatment (17/238 men from 10 studies).^35^ Whether or not these men received adjuvant therapy is unclear.

Luteinizing hormone is rarely reported in these series, but is important to consider as compensated hypogonadism is common in men with solitary testis, and is associated with serious long‐term health effects such as higher incidence of cardiovascular disease and deterioration of cognitive and physical function.^33^ In the few series that report LH, only 2 men were noted to have an increase in LH out of 41 postoperatively (Tables 1, 2).

By removing less testicular parenchyma without sacrificing oncological outcomes, especially in testicular lesions more likely to be benign, we can potentially avoid testosterone deficiency or compensated hypogonadism that are more often associated with radical orchiectomy. This decision can be pursued after an informed discussion with the patient, and is an approach that has been endorsed in small tumors, under specific circumstances, by multiple guidelines recently.^8^, ^36^

### 3.5 Semen parameters

Testicular cancer is the most common malignancy among men between the ages of 15 and 44, a timeframe that includes the prime reproductive window for most men who desire families.^37^ Therefore it is important to discuss the impact of fertility, and how it may be preserved using TSS, obviously with oncological safety as top priority. We identified 17 studies that reported on either paternity or sperm‐related outcomes (Table 3). In one series, six men found fertility important enough to delay adjuvant radiation in order to have families, with no worsening of oncological outcomes.^34^

**TABLE 3:** Current data available regarding fertility or semen parameters following TSS

First author (Year)	Benign/Malignant lesions	Adjuvant RT (Dose)	Sperm concentration (10^6^/ml)		Sperm motility (%)		Paternity status postoperative (Achieve/Attempt)
			Pre	Post	Pre	Post	
Pozza (2019)^39^	40/0	None	4.5 (0.1‐31.5)	3.0 (0.3‐33.0)	15.0 (0‐40.2)	15.5 (0‐28.7)	2/4^a^
Lagabrielle (2018)^64^	24/6	2 received EBR	15/30 Azo	15/30 Azo	NR	NR	1/1
			12/30 OAT	12/30 OAT			
Steiner (2003)^44^	18/12	18Gy in 8/30	NR	1/1 Normo	NR	NR	1/1
Carmignani (2007)^66^	20/2	None	NR	4/22 Azo	NR	NR	0/3
Hallak (2009)^24^	4/1	18Gy in 1/5	5/5 Azo	NR	0	0	1/5^b^
Liu (2015)^67^	9/2	None	139.2 ± 53.5	153.8 ± 28.5	44.5 ± 26	38.6 ± 11.6	NR
Claahsen‐van der Grinten (2007)^68^	6/0	None	5/6 Azo	5/6 Azoospermic	NR	NR	NR
			1/6 Oligo	1/6 Oligo			
Gentile (2013)^26^	13/2	None	9/9 Normal*	9/9 Normal*	9/9 Normal*	9/9 Normal*	NR
Leonhartsberger (2014)^22^	25/8	18Gy in 5/8	3/25 Azo	NR	NR	NR	NR
			2/25 SCO				
Heidenreich (2001)^47^	0/73	Yes, 46/73 18Gy	NR	NR	NR	NR	5/10^c^
Mearini (1996)^72^	0/1	None; PEB chemotherapy	0.7	3	NR	NR	NR
Bojanic (2015)^48^	1/25	None	20/22 Normo/Oligo	2/22 Azo	NR	NR	4/4^a^
			2/22 Azo				
Benelli (2017)^76^	13/1	None	2/5 Oligo	2/5 Oligo	NR	NR	5/5^a^
			2/5 OAT	2/5 OAT			
Ostergren (2017)^77^	1/1	None	1/1 Azo	NR	NR	NR	NR
Giannarini (2007)^16^	17/0	None	NR	NR	NR	NR	10/14
Rolle (2006)^23^	1/0	None	1/1 Azo	1/1 Azo	0	0	0/1^b^
Canda (2009)^78^	0/1	No	1/1 Azo	NR	0	NR	NR

^a^ Using Assisted reproductive technology (ART); Normo = Normozoospermia; Azo, Azoospermic; Oligo, Oligozoospermic; OAT, Oligoastehnoteratozoospermia; EBR, External Beam Radiotherapy; and SCO, Sertoli Cell Only;

^b^ Microdissection Testicular sperm extraction performed and available for cryopreservation;

^c^ Performed cryopreservation before TSS.

* Spermiogram only performed in patients younger than 40.

Unfortunately, most men undergoing surgery for benign or malignant testicular lesions have abnormal sperm parameters preoperatively (Table 3), a phenomemon well documented in the literature.^38^ From the limited data available on fertility post‐TSS, sperm parameters do not appear to change significantly. The largest trial of TSS to track sperm parameters in men having surgery for benign lesions found that most men were oligospermic and aesthenospermic preoperatively, with no significant decline postoperatively.^39^ This is in contrast to radical orchiectomy, where semen parameters inevitably worsen, even in the absence of adjuvant therapies.^2^, ^40^ A large‐scale comparison of these two groups on fertility has not yet been performed, however, in men with synchronous tumors or a tumor in a solitary testis, performing TSS is the only option for men to be able to attempt natural conception in the future. Regardless of the approach used, clinicians must keep fertility preservation guidelines in mind^41^ and discuss sperm cryopreservation prior to surgery, as this relatively simple intervention is still forgotten in the majority of cases for men.^42^

### 3.6 Oncological outcomes

The long‐life expectancy of testis cancer patients has prompted the urological community to explore a more conservative approach to patients who wish to avoid late adverse events derived from losing testicular function.^43^ However, radical orchiectomy is still considered the gold standard approach to testicular masses of suspicious or malignant origin.

The widespread use of ultrasonography has led to an increase in the number of incidentally detected small testicular masses.^4^, ^44^ Furthermore, the close follow‐up of patients treated with radical orchiectomy for testicular cancer has led to a rise in detection of small tumors in the contralateral testis,^45^ leaving TSS as an excellent option for preserving testicular function while maintaining adequate oncological outcome.^16^ We identified 12 studies that reported on TSS for malignant testicular tumors.

The first successful testis‐sparing surgery was performed by Richie, who performed the procedure for a synchronous bilateral seminoma. The patient remained free of disease without the need for permanent androgen replacement at 2.5 years follow‐up. The author himself described this management as “unorthodox”.^46^

Since then, several series, case reports, and systematic reviews have described TSS for selected patients with GCTs (organ‐confined tumors in patients with synchronous bilateral tumors or solitary testis with normal preoperative endocrine function).^16^

The largest series concerning TSS for malignant GCT was reported by The German Testicular Cancer Study Group. TSS was successfully performed in 101 patients with bilateral GCT, or solitary testis GCT. Surgery was performed at eight high volume institutions. Mean tumor diameter was 15 mm (5‐30 mm). Germ cell neoplasia in situ (GCNIS) was found in 84% of the cases and 79% underwent adjuvant radiation with 18 Gy. After a median follow‐up of 80 months, 100 patients remained with no evidence of disease. Local recurrence developed in two patients with associated GCNIS after local radiation and in four patients who postponed radiation for paternity reasons. All six patients were salvaged by inguinal orchiectomy.^34^, ^47^

Steiner et al reported TSS in 11 patients with GCT.^44^ All tumors were less than 25 mm in diameter, and 10 of them were diagnosed with concomitant ipsilateral GCNIS. All but two patients with GCNIS received local radiation with 18 Gy. One local recurrence was seen in a patient with GCNIS who decided not to undergo local radiation to preserve fertility; TSS was repeated and patient later gave consent to receive local radiation. All patients were free of disease at a mean follow‐up of 46.3 months.

Bojanic et al, reported 24 patients who underwent TSS for bilateral GCT or solitary testis tumors.^48^ Tumor size was <2 cm in all cases. A total of seven patients developed local recurrence, five of them had GCNIS and were salvaged with radical orchiectomy; a second TSS was done in the other two patients. At a median follow‐up of 51 months, overall survival of the study group was 100%.

The management of GCNIS is important in these cases because the majority of GCNIS cases will progress into invasive disease without treatment.^49^ The presence of GCNIS in a testis is associated with an estimated risk of developing invasive disease of 50% within 5 years and 70% within 7 years.^50^ In cases of biopsy‐proven GCNIS the cumulative probability for developing testicular cancer ranges between 30% and 70% after 7‐15 years.^51^

Petersen et al, analyzed the effect of radiotherapy for eradication of GCNIS in the testis.^52^ A total of 48 patients received local radiation at doses of 14, 16, 18, and 20 Gy. All patients treated at dose levels of 16 Gy‐20 Gy achieved histologically verified complete remission without signs of recurrence at 5 years follow‐up. One of the patients treated with 14 Gy had a relapse of GCNIS 20 months after radiation. These findings are reflected in the European Association of Urology 2020 Testicular Cancer Guidelines, which recommend offering local radiotherapy (18‐20 Gy in fractions of 2 Gy) for patients with GCNIS in a solitary testis.^53^ Fertile patients who wish to father a child may delay radiation, but close follow‐up with regular testicular US and clinical examination is mandatory.^16^

From these series, it appears that under the right circumstances, TSS for small testicular masses has a reasonable cure rate, with the ability to control GCNIS with adjuvant radiation, and perform a salvage orchiectomy in case of recurrence (Table 2). Follow‐up after TSS has not been well defined and has not been studied prospectively in any published literature. In fact, up to 21% of men are lost to follow‐up in these series. Thus patient selection and cautious, frequent follow‐up with integration of US is necessary until improved protocols are developed.^54^, ^55^ According to current guidelines, testis‐sparing surgery can be considered in patients with bilateral GCT or a solitary testis with a mass suspicious for GCT.^53^, ^56^ Expanding this indication to men with both testicles in situ at the time of surgery will require further research and controlled comparison with the gold standard radical orchiectomy.

### 3.7 Nonpalpable tumors

Increased use of scrotal US for orchialgia or infertility has led to increased detection rates for small, nonpalpable testicular masses.^57^, ^58^ Final histopathological examination concludes that 50%‐80% of incidentally detected lesions < 2 cm are benign, with Leydig cell tumors being the most common variant.^4^, ^8^, ^59^ Of 11 studies in this review with complete information, 229 (81%) of a total of 282 US‐detected testicular masses under 2 cm were found to be benign (Table 4). Gentile et al reported the largest series of TSS for nonpalpable tumors. Ninety‐one of 147 patients in this series presented with a nonpalpable tumor that was either found incidentally or as part of infertility workup.^60^ In these patients, preoperative tumor size was 8.7 mm with 76 of 91 tumors identified as benign on final histology. This series confirmed the predictive value of size on predicting malignancy, with a size cutoff of 8.5 mm having a 95% negative predictive value for malignant pathology.

**TABLE 4:** Series reporting on nonpalpable, ultrasound‐detected tumors

First author (Year)	(#) of nonpalpable tumors	US Tumor Size (mm) Median ± Std (Range)	Final pathology		Frozen section examination (FSE)		Conversions (where frozen section indicated benign, but final path indicated malignant)
			(#) Benign	(#) Malignant	PPV (%)	Sensitivity	
Lagabrielle (2018)^64^	32	8.5 (4.7 – 10)	24	8	92	80	1/32
Hallak (2009)^24^	6	14.8 ± 3.6	5	1	100	100	0/6
Egan (2020)^69^	14	NR	11	3	92	100	NR
Gentile (2013)^26^	10	7.6	9	1	80	NR	0/10
Lawrentschuck (2011)^70^	3	NR	3	0	100	100	0/3
Rolle (2006)^23^	7	5.7 ± 4.6 (2.5‐16)	6	1	100	100	NR
Mancini (2007)^27^	13	(2.8‐26)	9	2	NR	NR	NR
Dell’Atti (2016)^12^	20	11.6 (6‐15)	6	14	100	84.3	NR
De Stefani (2012)^28^	23	14.3 ± 5.2	21	2	100	100	0/23
Loeser (2009)^79^	7	NR	7	0	NR	83.3	0/7
Gentile (2020)^60^	91	9.2 ± 5.2 (5‐12)	126	21	24*	81*	0/147

* Using a cutoff of 8.5 mm lesion diameter.

Testis‐sparing surgery for nonpalpable tumors remains as an acceptable alternative, as many series report safe and effective oncologic and functional outcomes. Nonpalpable tumors are good candidates for TSS, as many have recently been managed with active surveillance with good outcomes. Bienek et al reported that close US surveillance appeared safe in a series of 120 infertile men diagnosed with testicular mass, only 18 (15%) of which underwent surgical exploration.^4^ The average mass size in this trial was 4.14 mm (±2 mm). Of those on active surveillance, an average follow‐up length of 1.3 years showed that the overall lesion growth rate was negligible. While active surveillance may avoid direct intervention, the active surveillance may place patients on an indefinite routine of follow‐up visits, creating an additional undue burden that may have been solved by surgical extirpation.

Despite the apparent safety of TSS in small, US‐detected lesions, there still remains a small chance for malignancy. Muller et al reported a series of 20 men who underwent surgical exploration for a nonpalpable tumor < 5 mm, 4 of which were found to have GCNIS.^61^ In another series by Khan et al, 3 of 12 patients that presented with issues related to infertility were found to have an incidental mass on scrotal US.^62^ In the first patient, frozen section examination (FSE) showed a high‐grade B cell lymphoma. Thus, TSS with FSE may be considered as an initial approach for patients with nonpalpable tumors, with the understanding that a radical orchiectomy may be necessary given the possibility for malignancy.

## 4 CONCLUSION

**FIGURE 3:**
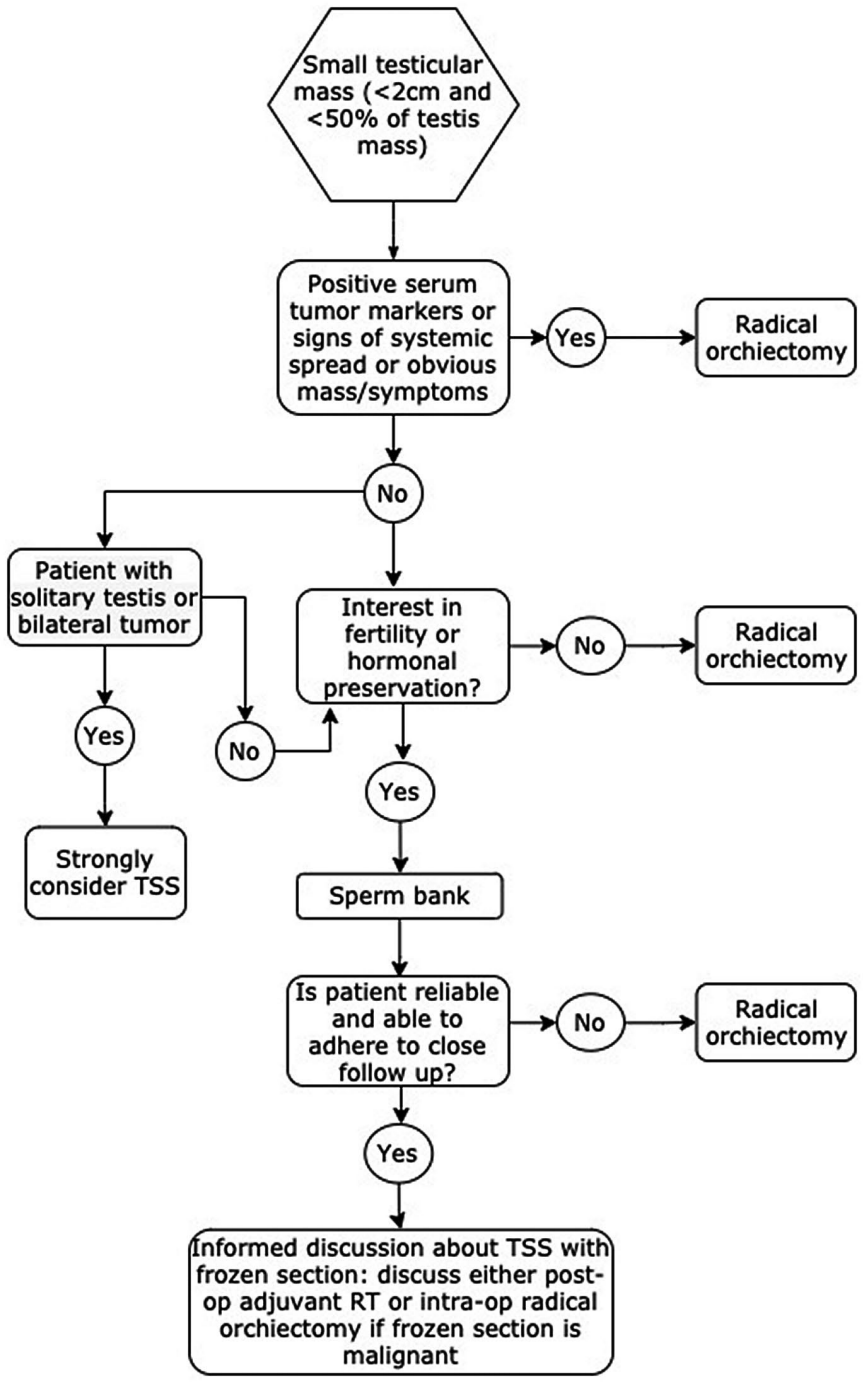
Algorithm to follow for management of a small testis mass

Men with malignant and benign testicular tumors suffer from underlying spermatogenic failure and are at risk for postoperative testosterone deficiency following radical orchiectomy. Testis‐sparing surgery is an option to preserve function in men with a testicular mass. Figure 3 outlines a basic algorithm for approaching these cases.

Guidelines support pursuing TSS in men where radical orchiectomy would leave them anorchid, or in circumstances where fertility or hormone production would be seriously compromised. Among men with small lesions, TSS is a reasonable option, as transitioning to radical orchiectomy at the time of frozen section should not compromise outcomes, and adjuvant treatment in the context of GCNIS shows excellent rates of oncological control. As no standardized protocols for postoperative follow‐up after TSS have been investigated, an abundance of caution and frequent clinical visits are warranted. Testis‐sparing surgery with or without use of operative microscope should be in the armamentarium of urologists so patients can be guided with a shared decision‐making approach.

## CONFLICT OF INTEREST

JO, UB, DCG, AAS, JTW, CDR, KJ, and JR have no conflict of interest to report. RR has no conflict of interest relevant to this project. RR is a consultant for: Acerus pharmaceuticals, Boston Scientific, Coloplast, Endo Pharmaceuticals, Metuchen, and Nestle Health. RR is a grant recipient from: Boston Scientific, Coloplast, and Endo Pharmaceuticals.

## AUTHOR CONTRIBUTIONS

Study design JO, UB, and JR. Study conduct: RR and KJ. Data collection: DCG, AS, CDR, JTW, and JR. Data interpretation: JO and UB. Drafting manuscript: JO, UB, DCG, AS, CDR, JTW, and JR. Revising manuscript content: JO, UB, KJ, and RR. Approving final version of manuscript: KJ and RR.

## Supporting information

Supplementary Material
