# Supplementary material for: Outcomes of organ‐sparing surgery for adult testicular tumors: A systematic review of the literature
Source: BJUI Compass. 2021 Feb 23;2(5):306–21. doi: 10.1002/bco2.77 (PMC8462801; doi:10.1002/bco2.77)
Supplement: Supplementary file 1 — Supplementary Material [file BCO2-2-306-s001.docx]

**Appendix - Database Search Strategies**

**Ovid MEDLINE(R) and Epub Ahead of Print, In-Process & Other Non-Indexed Citations and Daily <1946 to November 20, 2020>
Searched November 20, 2020**

| 1 | [https://orcid.org/0000-0001-6744-3517] |
| --- | --- |
| 2 | [testicular tumors terms] |
| 3 | testicular neoplasms/ or sertoli-leydig cell tumor/ or leydig cell tumor/ or sertoli cell tumor/ |
| 4 | "neoplasms, germ cell and embryonal"/ or carcinoma, embryonal/ or germinoma/ or seminoma/ or gonadoblastoma/ or endodermal sinus tumor/ or teratocarcinoma/ |
| 5 | ((testes or testicle* or testicu* or testis or gonad or gonads or gonadal or sertoli-leydig or leydig or leydig-cell or sertoli or sertoli-cell) adj5 (adenoma* or cancer* or carcinoma* or lesion* or malignan* or mass or neoplas* or sarcoma* or tumo?r*)).ti,ab,kf. |
| 6 | (androblastoma* or arrhenoblastoma* or tubular adenoma*).ti,ab,kf. |
| 7 | ((germ-cell* or germinal* or embryon*) adj3 (adenoma* or cancer* or carcinoma* or lesion* or malignan* or mass or neoplas* or sarcoma* or tumo?r*)).ti,ab,kf. |
| 8 | (germinoma or germinomas or seminoma or seminomas or spermatocytoma or spermatocytomas or spermocytoma or spermocytomas).ti,ab,kf. |
| 9 | (gonadoblastoma or gonadoblastomas or dysgenetic gonadoma*).ti,ab,kf. |
| 10 | (((endodermal-sinus or yolk-sac or yolk-sack) adj3 (adenoma* or cancer* or carcinoma* or lesion* or malignan* or mass or neoplas* or sarcoma* or tumo?r*)) or orchioblastoma*).ti,ab,kf. |
| 11 | 3 or 4 or 5 or 6 or 7 or 8 or 9 or 10 |
| 12 | [testicular sparing surgery terms] |
| 13 | Orchiectomy/ |
| 14 | (spar* or saving or preserv* or conserv* or enucleat* or resect*).ti,ab. |
| 15 | 13 and 14 |
| 16 | ((orchidectom* or orchiectom*) adj3 (partial or spar* or saving or preserv*)).ti,ab,kf. |
| 17 | (hemiorchiectom* or semiorchiectom* or semi-orchiectom* or hemi-orchiectom*).ti,ab,kf. |
| 18 | ((spar* or saving or preserv* or conserv* or enucleat* or resect*) adj7 (gonad* or testes or testi* or organ)).ti,ab,kf. |
| 19 | ((conserv* adj3 (manag* or surg* or approach*)) and (gonad* or testes or testi* or organ)).ti,ab,kf. |
| 20 | 15 or 16 or 17 or 18 or 19 |
| 21 | 11 and 20 |
| 22 | [filters for species and study type] |
| 23 | 21 not ((exp animal/ or exp invertebrate/ or animal experiment/ or animal model/ or exp plant/ or exp fungus/) not exp human/) |
| 24 | 23 not ((review or case reports).pt. or (case adj (report or study)).ti.) |

**Embase (Elsevier Embase.com)
Searched November 20, 2020.**Sources: Embase, Embase Classic, MEDLINE

Query: ('testis tumor'/exp OR 'germ cell cancer'/de OR 'germ cell carcinoma'/de OR 'embryonal carcinoma'/de OR 'teratocarcinoma'/de OR 'gonad tumor'/de OR 'gonadal cell tumor'/de OR 'gonadoblastoma'/de OR 'yolk sac tumor'/de OR (((testes OR testicle* OR testicu* OR testis OR gonad OR gonads OR gonadal OR 'sertoli leydig' OR leydig OR 'leydig cell' OR sertoli OR 'sertoli cell') NEAR/5 (adenoma* OR cancer* OR carcinoma* OR lesion* OR malignan* OR mass OR neoplas* OR sarcoma* OR tumo$r*)):ti,ab,kw) OR androblastoma*:ti,ab,kw OR arrhenoblastoma*:ti,ab,kw OR 'tubular adenoma*':ti,ab,kw OR ((('germ cell*' OR germinal* OR embryon*) NEAR/3 (adenoma* OR cancer* OR carcinoma* OR lesion* OR malignan* OR mass OR neoplas* OR sarcoma* OR tumo$r*)):ti,ab,kw) OR germinoma:ti,ab,kw OR germinomas:ti,ab,kw OR seminoma:ti,ab,kw OR seminomas:ti,ab,kw OR spermatocytoma:ti,ab,kw OR spermatocytomas:ti,ab,kw OR spermocytoma:ti,ab,kw OR spermocytomas:ti,ab,kw OR gonadoblastoma:ti,ab,kw OR gonadoblastomas:ti,ab,kw OR 'dysgenetic gonadoma*':ti,ab,kw OR ((('endodermal sinus' OR 'yolk sac' OR 'yolk sack') NEAR/3 (adenoma* OR cancer* OR carcinoma* OR lesion* OR malignan* OR mass OR neoplas* OR sarcoma* OR tumo$r*)):ti,ab,kw) OR orchioblastoma*:ti,ab,kw) AND ('orchiectomy'/de AND (spar*:ti,ab OR saving:ti,ab OR preserv*:ti,ab OR conserv*:ti,ab OR enucleat*:ti,ab OR resect*:ti,ab) OR (((orchidectom* OR orchiectom*) NEAR/3 (partial OR spar* OR saving OR preserv*)):ti,ab,kw) OR hemiorchiectom*:ti,ab,kw OR semiorchiectom*:ti,ab,kw OR 'semi orchiectom*':ti,ab,kw OR 'hemi orchiectom*':ti,ab,kw OR (((spar* OR saving OR preserv* OR conserv* OR enucleat* OR resect*) NEAR/7 (gonad* OR testes OR testi* OR organ)):ti,ab,kw) OR (((conserv* NEAR/3 (manag* OR surg* OR approach*)):ti,ab,kw) AND (gonad*:ti,ab,kw OR testes:ti,ab,kw OR testi*:ti,ab,kw OR organ:ti,ab,kw))) NOT ((rat:ti OR rats:ti OR mouse:ti OR mice:ti OR swine:ti OR porcine:ti OR murine:ti OR sheep:ti OR lambs:ti OR pigs:ti OR piglets:ti OR rabbit:ti OR rabbits:ti OR cat:ti OR cats:ti OR dog:ti OR dogs:ti OR cattle:ti OR bovine:ti OR monkey:ti OR monkeys:ti OR trout:ti OR marmoset$:ti) AND 'animal experiment'/de OR ('animal experiment'/de NOT ('human experiment'/de OR 'human'/de))) NOT ('chapter'/it OR 'note'/it OR 'review'/it OR 'case report'/de OR 'case report*':ti)

**Cochrane Central Register of Controlled Trials: CENTRAL (Wiley)
Searched November 20, 2020**

| ID | Search |
| --- | --- |
| #1 | [mh ^"testicular neoplasms"] OR [mh ^"sertoli-leydig cell tumor"] OR [mh ^"leydig cell tumor"] OR [mh ^"sertoli cell tumor"] |
| #2 | [mh ^"neoplasms, germ cell and embryonal"] OR [mh ^"carcinoma, embryonal"] OR [mh ^germinoma] OR [mh ^seminoma] OR [mh ^gonadoblastoma] OR [mh ^"endodermal sinus tumor"] OR [mh ^teratocarcinoma] |
| #3 | ((testes OR testicle* OR testicu* OR testis OR gonad OR gonads OR gonadal OR sertoli-leydig OR leydig OR leydig-cell OR sertoli OR sertoli-cell) NEAR/5 (adenoma* OR cancer* OR carcinoma* OR lesion* OR malignan* OR mass OR neoplas* OR sarcoma* OR tumo?r*)):ti,ab,kw |
| #4 | (androblastoma* OR arrhenoblastoma* OR "tubular adenoma*"):ti,ab,kw |
| #5 | ((germ-cell* OR germinal* OR embryon*) NEAR/3 (adenoma* OR cancer* OR carcinoma* OR lesion* OR malignan* OR mass OR neoplas* OR sarcoma* OR tumo?r*)):ti,ab,kw |
| #6 | (germinoma OR germinomas OR seminoma OR seminomas OR spermatocytoma OR spermatocytomas OR spermocytoma OR spermocytomas):ti,ab,kw |
| #7 | (gonadoblastoma OR gonadoblastomas OR (dysgenetic NEXT gonadoma*)):ti,ab,kw |
| #8 | (((endodermal-sinus OR yolk-sac OR yolk-sack) NEAR/3 (adenoma* OR cancer* OR carcinoma* OR lesion* OR malignan* OR mass OR neoplas* OR sarcoma* OR tumo?r*)) OR orchioblastoma*):ti,ab,kw |
| #9 | {or #1-#8} |
| #10 | [mh ^Orchiectomy] |
| #11 | (spar* OR saving OR preserv* OR conserv* OR enucleat* OR resect*):ti,ab |
| #12 | #10 AND #11 |
| #13 | ((orchidectom* OR orchiectom*) NEAR/3 (partial OR spar* OR saving OR preserv*)):ti,ab,kw |
| #14 | (semi?orchiectom* OR hemi?orchiectom*):ti,ab,kw |
| #15 | ((spar* OR saving OR preserv* OR conserv* OR enucleat* OR resect*) NEAR/7 (gonad* OR testes OR testi* OR organ)):ti,ab,kw |
| #16 | ((conserv* NEAR/3 (manag* OR surg* OR approach*)) AND (gonad* OR testes OR testi* OR organ)):ti,ab,kw |
| #17 | {or #12-#16} |
| #18 | #9 AND #17 in Trials |

**CINAHL Plus with Full Text (Ebsco)
Searched November 20, 2020**

| S19 | S18 NOT ((PT review) OR (PT meta analysis) or ( PT systematic review) OR (PT case study) OR (TI case N0 study) OR (TI case N0 report)) |
| --- | --- |
| S18 | s17 NOT (MH "Animals+" NOT MH "Human") |
| S17 | S8 AND S16 |
| S16 | S11 OR S12 OR S13 OR S14 OR S15 |
| S15 | (((TI conserv* OR AB conserv*) N3 ((TI manag* OR AB manag*) OR (TI surg* OR AB surg*) OR (TI approach* OR AB approach*))) AND ((TI gonad* OR AB gonad*) OR (TI testes OR AB testes) OR (TI testi* OR AB testi*) OR (TI organ OR AB organ))) |
| S14 | (((TI spar* OR AB spar*) OR (TI saving OR AB saving) OR (TI preserv* OR AB preserv*) OR (TI conserv* OR AB conserv*) OR (TI enucleat* OR AB enucleat*) OR (TI resect* OR AB resect*)) N7 ((TI gonad* OR AB gonad*) OR (TI testes OR AB testes) OR (TI testi* OR AB testi*) OR (TI organ OR AB organ))) |
| S13 | ((TI hemiorchiectom* OR AB hemiorchiectom*) OR (TI semiorchiectom* OR AB semiorchiectom*) OR (TI semi-orchiectom* OR AB semi-orchiectom*) OR (TI hemi-orchiectom* OR AB hemi-orchiectom*) OR (TI hemi-orchiectom* OR AB hemi-orchiectom*)) |
| S12 | (((TI orchidectom* OR AB orchidectom*) OR (TI orchiectom* OR AB orchiectom*)) N3 ((TI partial OR AB partial) OR (TI spar* OR AB spar*) OR (TI saving OR AB saving) OR (TI preserv* OR AB preserv*))) |
| S11 | S9 AND S10 |
| S10 | ((TI spar* OR AB spar*) OR (TI saving OR AB saving) OR (TI preserv* OR AB preserv*) OR (TI conserv* OR AB conserv*) OR (TI enucleat* OR AB enucleat*) OR (TI resect* OR AB resect*)) |
| S9 | (MH "Orchiectomy") |
| S8 | S1 OR S2 OR S3 OR S4 OR S5 OR S6 OR S7 |
| S7 | ((((TI endodermal-sinus OR AB endodermal-sinus) OR (TI yolk-sac OR AB yolk-sac) OR (TI yolk-sack OR AB yolk-sack)) N3 ((TI adenoma* OR AB adenoma*) OR (TI cancer* OR AB cancer*) OR (TI carcinoma* OR AB carcinoma*) OR (TI lesion* OR AB lesion*) OR (TI malignan* OR AB malignan*) OR (TI mass OR AB mass) OR (TI neoplas* OR AB neoplas*) OR (TI sarcoma* OR AB sarcoma*) OR (TI tumo#r* OR AB tumo#r*))) OR (TI orchioblastoma* OR AB orchioblastoma*)) |
| S6 | ((TI gonadoblastoma OR AB gonadoblastoma) OR (TI gonadoblastomas OR AB gonadoblastomas) OR (TI "dysgenetic gonadoma*" OR AB "dysgenetic gonadoma*")) |
| S5 | ((TI germinoma OR AB germinoma) OR (TI germinomas OR AB germinomas) OR (TI seminoma OR AB seminoma) OR (TI seminomas OR AB seminomas) OR (TI spermatocytoma OR AB spermatocytoma) OR (TI spermatocytomas OR AB spermatocytomas) OR (TI spermocytoma OR AB spermocytoma) OR (TI spermocytomas OR AB spermocytomas)) |
| S4 | (((TI germ-cell* OR AB germ-cell*) OR (TI germinal* OR AB germinal*) OR (TI embryon* OR AB embryon*)) N3 ((TI adenoma* OR AB adenoma*) OR (TI cancer* OR AB cancer*) OR (TI carcinoma* OR AB carcinoma*) OR (TI lesion* OR AB lesion*) OR (TI malignan* OR AB malignan*) OR (TI mass OR AB mass) OR (TI neoplas* OR AB neoplas*) OR (TI sarcoma* OR AB sarcoma*) OR (TI tumo#r* OR AB tumo#r*))) |
| S3 | ((TI androblastoma* OR AB androblastoma*) OR (TI arrhenoblastoma* OR AB arrhenoblastoma*) OR (TI "tubular adenoma*" OR AB "tubular adenoma*")) |
| S2 | (((TI testes OR AB testes) OR (TI testicle* OR AB testicle*) OR (TI testicu* OR AB testicu*) OR (TI testis OR AB testis) OR (TI gonad OR AB gonad) OR (TI gonads OR AB gonads) OR (TI gonadal OR AB gonadal) OR (TI sertoli-leydig OR AB sertoli-leydig) OR (TI leydig OR AB leydig) OR (TI leydig-cell OR AB leydig-cell) OR (TI sertoli OR AB sertoli) OR (TI sertoli-cell OR AB sertoli-cell)) N5 ((TI adenoma* OR AB adenoma*) OR (TI cancer* OR AB cancer*) OR (TI carcinoma* OR AB carcinoma*) OR (TI lesion* OR AB lesion*) OR (TI malignan* OR AB malignan*) OR (TI mass OR AB mass) OR (TI neoplas* OR AB neoplas*) OR (TI sarcoma* OR AB sarcoma*) OR (TI tumo#r* OR AB tumo#r*))) |
| S1 | (MH "Testicular Neoplasms") OR (MH "Neoplasms, Germ Cell and Embryonal") OR (MH "Germinoma") OR (MH "Seminoma") |

**Scopus (Elsevier)
Searched November 20, 2020.**

((TITLE-ABS-KEY( ( ((testes or testicle* or testicu* or testis or gonad or gonads or gonadal or sertoli-leydig or leydig or leydig-cell or sertoli or sertoli-cell) W/5 (adenoma* or cancer* or carcinoma* or lesion* or malignan* or mass or neoplas* or sarcoma* or tumo?r*)) OR androblastoma* or arrhenoblastoma* or "tubular adenoma" OR germinoma or germinomas or seminoma or seminomas or spermatocytoma or spermatocytomas or spermocytoma or spermocytomas OR orchioblastoma* OR ((germ-cell* or germinal* or embryon*) W/3 (adenoma* or cancer* or carcinoma* or lesion* or malignan* or mass or neoplas* or sarcoma* or tumor*)) OR ((endodermal-sinus or yolk-sac or yolk-sack) W/3 (adenoma* or cancer* or carcinoma* or lesion* or malignan* or mass or neoplas* or sarcoma* or tumor*)) ) AND ( ((orchidectom* or orchiectom*) W/3 (partial or spar* or saving or preserv*)) OR hemiorchiectom* or semiorchiectom* or semi-orchiectom* or hemi-orchiectom* OR ((spar* or saving or preserv* or conserv* or enucleat* or resect*) W/7 (gonad* or testes or testi* or organ)) OR ((conserv* W/3 (manag* or surg* or approach*)) AND (gonad* or testes or testi* or organ)) ) )) AND NOT (((DOCTYPE("review") OR DOCTYPE("case reports")) OR (TITLE("case") W/1 (TITLE("report") OR TITLE("study")))))) AND NOT (TITLE ((animal* OR rat OR rats OR mouse OR mice OR murine OR dog OR dogs OR canine OR cat OR cats OR feline OR rabbit OR cow OR cows OR bovine OR rodent* OR sheep OR ovine OR pig OR swine OR porcine OR veterinar* OR chick* OR zebrafish* OR baboon* OR nonhuman* OR primate* OR cattle* OR goose OR geese OR duck OR macaque* OR avian* OR bird* OR fish* OR trout* or marmoset* or chimp*) AND NOT (human* OR patient* OR women OR woman OR men OR man))) AND ( EXCLUDE ( SRCTYPE,"b" ) OR EXCLUDE ( SRCTYPE,"k" ) )

**Web of Science (Clarivate)
Searched November 20, 2020.**

Web of Science Core Collection:

Science Citation Index Expanded (SCI-EXPANDED) --1945-present

Social Sciences Citation Index (SSCI) --1956-present

Arts & Humanities Citation Index (A&HCI) --1975-present

Conference Proceedings Citation Index- Science (CPCI-S) --1990-present

Conference Proceedings Citation Index- Social Science & Humanities (CPCI-SSH) --1990-present

Emerging Sources Citation Index (ESCI) --2015-present

plus:
KCI-Korean Journal Database 1980-present
Russian Science Citation Index 2005 – present
SciELO Citation Index 2002 – present
Timespan=1945-2020,Language = Auto

TS=( (((testes OR testicle* OR testicu* OR testis OR gonad OR gonads OR gonadal OR sertoli-leydig OR leydig OR leydig-cell OR sertoli OR sertoli-cell) NEAR/5 (adenoma* OR cancer* OR carcinoma* OR lesion* OR malignan* OR mass OR neoplas* OR sarcoma* OR tumo$r*) ) OR (androblastoma* OR arrhenoblastoma* OR "tubular adenoma*") OR ((germ-cell* OR germinal* OR embryon*) NEAR/3 (adenoma* OR cancer* OR carcinoma* OR lesion* OR malignan* OR mass OR neoplas* OR sarcoma* OR tumo$r*) ) OR (germinoma OR germinomas OR seminoma OR seminomas OR spermatocytoma OR spermatocytomas OR spermocytoma OR spermocytomas) OR (gonadoblastoma OR gonadoblastomas OR "dysgenetic gonadoma*") OR (((endodermal-sinus OR yolk-sac OR yolk-sack) NEAR/3 (adenoma* OR cancer* OR carcinoma* OR lesion* OR malignan* OR mass OR neoplas* OR sarcoma* OR tumo$r*) ) OR orchioblastoma*)) AND (((orchidectom* OR orchiectom*) NEAR/3 (partial OR spar* OR saving OR preserv*) ) OR (hemiorchiectom* OR semiorchiectom* OR semi-orchiectom* OR hemi-orchiectom* OR hemi-orchiectom*) ((spar* OR saving OR preserv* OR conserv* OR enucleat* OR resect*) NEAR/7 (gonad* OR testes OR testi* OR organ) ) OR ((conserv* NEAR/3 (manag* OR surg* OR approach*) ) AND (gonad* OR testes OR testi* OR organ) ) OR ((Orchiectomy) AND ((spar* OR saving OR preserv* OR conserv* OR enucleat* OR resect*) )) OR (((orchidectom* OR orchiectom*) NEAR/3 (partial OR spar* OR saving OR preserv*) )) OR ((hemiorchiectom* OR semiorchiectom* OR semi-orchiectom* OR hemi-orchiectom* OR hemi-orchiectom*) ) OR (((spar* OR saving OR preserv* OR conserv* OR enucleat* OR resect*) NEAR/7 (gonad* OR testes OR testi* OR organ) )) OR (((conserv* NEAR/3 (manag* OR surg* OR approach*) ) AND (gonad* OR testes OR testi* OR organ) )))) NOT (( TS= (animal* OR rat OR rats OR mouse OR mice OR murine OR dog OR dogs OR canine OR cat OR cats OR feline OR rabbit OR cow OR cows OR bovine OR rodent* OR sheep OR ovine OR pig OR swine OR porcine OR veterinar* OR chick* OR zebrafish* OR baboon* OR nonhuman* OR primate* OR cattle* OR goose OR geese OR duck OR macaque* OR avian* OR bird* OR fish* OR trout* or marmoset* or chimp*) ) OR (DT=(CASE REPORT) OR TI=("case report*") OR TI=("case stud*") ))

**Clinicaltrials.gov (U.S. National Libraries of Medicine)
Searched November 20, 2020**

surgery | Studies With Results | Testicular Cancer
surgery | Studies With Results | Testicular Neoplasms
surgery | Studies With Results | Testicular Tumor

**World Health Organization International Clinical Trials Registry Platform (https://www.who.int/ictrp/en)
Searched November 20, 2020**

testic* and surger* - with synonyms, limit to studies with results only

**John Reynolds, MLIS, AHIP
Calder Memorial Library
University of Miami Miller School of Medicine
Miami,FL**

**https://orcid.org/0000-0001-6744-3517**

**Original Developmental Search Strategy
Searched October, 2020 by J. Ory and D. Gonzalez**

**PubMed (National Library of Medicine)**

(((((testi* sparing) OR (preserving surgery)) OR (radical orchidectomy)) AND (fertility)) AND (testi* lesion))

**Scopus (Elsevier)**

TITLE-ABS-KEY ( ( ( ( ( ( testi* AND sparing ) OR ( preserving AND surgery ) ) OR ( radical AND orchidectomy ) ) AND ( fertility ) ) AND ( testi* AND lesion ) ) )
